# Supplementary material for: Specific pelvic shape in patients with developmental dysplasia of the hip on 3D morphometric homologous model analysis
Source: PLoS One. 2024 Jun 3;19(6):e0300938. doi: 10.1371/journal.pone.0300938 (PMC11146717; doi:10.1371/journal.pone.0300938)
Supplement: Attachment — Submitted filename: Response to Reviewers.docx [file pone.0300938.s002.docx]

Response to Reviewers

The authors would like to thank the reviewers and editors for their time and effort to review this manuscript.

Reviewer #1

The introduction section please state the aim of your study.

The aim of our study added to introduction.

The methods and the results section is well-written but please add comparative data like Sharp angle, sacral slope and other measurements between normal and DDH group.

Measurement values such as Sharp angle etc. are shown in the table 2.

In discussion section, at first paragraph please remove "Although morphological analysis using a homologous model assumes that all vertices comprising the pelvis are homologous, it is an objective method that uses all morphological information. This method has recently been used to evaluate foot form, cranial morphology, facial aging changes, and pelvis sex determination, and it is attracting attention as a new comparison method for 3D models [15, 19, 21, 24]" and move to next or further paragraph.

In the first paragraph clearly state the most important findings of your study.

The sentences you pointed out were moved to the second paragraph.

Reviewer #2:

In the title of the article, it is stated that specific pelvic shapes in patients with developmental hip dysplasia were examined by model analysis. However, it is stated that all DDH patients used in the modeling underwent rotational osteotomy surgery. In fact, these patients should be stated as DDH patients who underwent surgery. The age range of this patient group is given as 21-52 years. It should be stated at what age the patients had surgery and how long after the surgery the modeling was done.

The 3D pelvic models of a DDH patients were reconstructed from CT images before rotational acetabular osteotomy. We added this information on page 5, lines 88-90.
